# A Taste Bud Organoid-Based Biosensor with a 3-Dimensional Microelectrode Array for Evaluating Caffeine’s Impacts on Taste Sensing

**DOI:** 10.34133/bmef.0286

**Published:** 2026-07-02

**Authors:** Shuge Liu, Yuqi Chen, Zhiyao Wang, Miaomiao Wang, Yating Chen, Yulan Tian, Xinyi Liu, Jingyi Li, Jingxi Li, Liping Du, Xiaojun Li, Chunsheng Wu

**Affiliations:** ^1^ Department of Ophthalmology, The Second Affiliated Hospital of Xi’an Jiaotong University, Xi’an 710004, China.; ^2^ Institute of Light Resources and Environmental Sciences, Henan Academy of Sciences, Zhengzhou 450046, China.; ^3^ Institute of Medical Engineering, Department of Biophysics, School of Basic Medical Sciences, Health Science Center, Xi’an Jiaotong University, Xi’an 710061, China.; ^4^ Department of Biomedical Engineering, Air Force Medical University, Xi’an 710032, China.; ^5^ Frontier Institute of Science and Technology, Xi’an Jiaotong University, Xi’an 710049, China.

## Abstract

**Objective:** The impact of caffeine on the gustatory system is still not fully understood. Therefore, it is highly essential to investigate caffeine’s impacts on taste sensing. **Impact Statement:** The present study develops a taste bud organoid-based biosensor using a 3-dimensional microelectrode array to systematically investigate the modulatory effects of caffeine on various taste perceptions. **Introduction:** The integration of taste bud organoids with a 3-dimensional microelectrode array introduces a novel class of biomimetic taste sensors. This study utilizes this sophisticated sensor technology to systematically assess the responses of caffeine-treated taste bud organoids to a range of flavor stimuli. **Methods:** Biosensor functionality was assessed through metrics such as the proportion of responsive electrodes and signal-to-noise ratio. The electrophysiological characterization of the firing patterns was analyzed including firing rate and amplitude, in response to diverse flavor stimuli and caffeine exposure. Principal component analysis was employed to determine the sensor’s efficacy in identifying and differentiating distinct flavor profiles. **Results:** The results indicate that the electrode response rate of the fabricated chip ranges between 45% and 56%, with signal-to-noise ratio values ranging from 20.71 to 23.62. Taste bud organoids exhibit distinct electrophysiological responses contingent upon the taste stimulus: sweet stimuli elicit the strongest response, followed by sour stimuli, whereas responses to bitter, salty, and umami stimuli approximate baseline levels observed prior to stimulation. **Conclusion:** This investigation enhances the understanding of caffeine’s interaction with taste bud organoids, thereby contributing to the field of sensory biology and facilitating the advancement of sophisticated flavor sensing technologies.

## Introduction

In 2005, Tønning et al. [1] pioneered the development of biosensors designed to mimic the taste responses of biological systems in vitro. The biomimetic sensing and detection technology comprises 2 primary components: sensitive elements, which encompass taste bioactive substances such as receptors, cells [2], tissues [3], and biological taste systems [4], and transducers, which are fabricated using micro- and nanoscale devices [5–7]. Transducer arrays play a critical role in detecting and acquiring information regarding taste responses elicited by various substances. They process signals originating from the sensitive elements and relay the analyzed data to a pattern recognition module for final interpretation [8,9]. In contrast to traditional methods that employ electrochemical detector arrays, this novel approach is based on a well-defined principle of taste detection. Specifically, when a cell generates an action potential or exhibits synaptic activity, transmembrane ionic currents—such as those involving sodium (Na^+^) and potassium (K^+^) ions—alter the distribution of ionic concentrations within the local extracellular fluid. This alteration leads to the generation of a small potential difference, which can be measured and interpreted as a taste signal [10]. The microelectrode surface facilitates the transduction of ionic currents into measurable electrical signals through redox reactions. This surface not only captures the detection characteristics inherent to biological taste but also serves as a viable alternative to traditional chemical and physical sensors. Consequently, it enhances the biomimicry of taste perception and enables simpler and more accurate discrimination and detection of taste stimuli [10].

Currently, this advanced technology is extensively applied across diverse domains, including food safety—particularly in freshness evaluation [11], raw material identification [12], and taste analysis [13]—as well as in environmental monitoring [14] and pharmaceutical testing [15]. Accordingly, biosensors have become indispensable tools in taste detection. With the ongoing advancements in genetic engineering, bioactive materials, micro/nano detection techniques, neural coding and decoding mechanisms, brain–computer interfaces, and motion control systems, biosensing technologies are increasingly accessible. This progress is expected to provide powerful tools for the discovery of novel taste substances, early disease warning and prevention, improving social security, and the accumulation of ecological gustatory information within big data frameworks.

Organoid technology has undergone substantial advancement, evolving from mere structural biomimicry to the functional simulation of biological systems. This progression has been facilitated by pivotal developments in vascularization [16], maintenance of the microenvironment maintenance [17], and intelligent regulatory mechanisms [18]. Utilizing a lineage tracing and culture system, researchers have demonstrated that Lgr5-positive taste stem cells isolated from mouse tongue papillae can be effectively co-cultured with other taste stem cells using a matrix gel embedding technique. This novel method leads to the formation of taste bud organoids exhibiting 3-dimensional (3D) cellular architectures. These organoids display capabilities for self-renewal and differentiation, sustain the expression of taste receptors, and comprise mature taste receptor cells, including type II cells responsive to sweet, bitter, and umami stimuli, as well as type III cells sensitive to acidic tastes [19]. Subsequently, Adpaikar et al. [20] introduced a scaffold-free suspension culture methodology aimed at optimizing taste bud organoid development through the modulation of basal polarity. This technique enables precise spatial organization of taste receptor cells within the organoids.

Taste bud organoids have catalyzed marked progress across multiple disciplines, such as disease modeling, regenerative medicine, and biosensing. For example, exposure of these organoids to lipopolysaccharide induces the expression of inflammatory mediators, including tumor necrosis factor-alpha and interleukin-6, effectively recapitulating taste dysfunctions associated with radiotherapy or infectious processes. This provides a valuable in vitro model for elucidating the pathophysiological mechanisms underlying such disorders [21,22]. Moreover, RNA sequencing analyses have identified critical genes, such as Tas2r126, that play essential roles in taste cell differentiation, thereby offering novel perspectives for investigating gene-regulated taste pathologies [23]. In regenerative medicine, taste bud organoids cultured in suspension have been successfully transplanted into the epithelial tissue of murine tongues, leading to the restoration of neural innervation and recovery of gustatory function [20]. This promising strategy underscores the therapeutic potential of organoid technology for tissue repair and replacement.

Furthermore, the integration of microelectrode array (MEA) chips with taste bud organoids has facilitated the real-time recording of electrical signals elicited by diverse taste stimuli. This technological innovation importantly broadens the application scope of taste bud organoids within biosensing, enabling more refined and accurate gustatory detection methodologies [4,24]. Collectively, these advancements underscore the profound impact of taste bud organoids across various scientific and clinical domains.

Within the broader context of organoid research, 3D MEA systems have been effectively utilized for functional analyses of neural and cardiac organoids. However, their application to taste bud organoids remains relatively underexplored and is in nascent stages. Compared with 2-dimensional MEA chips, the advantages of 3D MEA chips lie in their ability to perform more physiologically relevant, comprehensive electrophysiological recording of organoids [4,25]. First, 3D MEA chips overcome the limitation of 2-dimensional chips that only capture signals from the basal surface, which could achieve full-structure coverage through spatial mapping, thus allowing for the recording of electrical signals from the interior and different depths of organoids. Second, the flexible and biomimetic design of 3D MEA chips could prevent the flattening of organoids caused by rigid substrates, thereby preserving the tissue’s native structure and ensuring physiological relevance and long-term stability of the data. Finally, 3D MEA chips allow for more precise analysis of complex functional connections, which could provide high-quality, standardized data support for many applications such as drug evaluation and disease modeling.

In the present study, we introduce a novel class of biomimetic taste sensors employing 3D MEA chips, which have been integratively incorporated into a biological taste detection platform to better match the 3D structure of taste organoids. Utilizing this sophisticated sensor technology, we systematically assess the responses of caffeine-treated taste bud organoids to a range of flavor stimuli. This approach enables precise discrimination and quantitative comparison of the 5 basic taste modalities, thereby enhancing our understanding of gustatory perception and demonstrating the potential utility of 3D MEA systems in taste bud organoid research. This work not only highlights the applicability of 3D MEA chips for taste detection but also lays the groundwork for future investigations into the functional characteristics of taste bud organoids.

## Results and Discussion

### Fabrication, modification, and characterization of the 3D MEA

The overall design of the chip features a square footprint measuring 49 × 49 mm, bordered by square contact pads each with dimensions of 1 mm × 1 mm to facilitate connections with external devices. This dimension represents a compromise between the chip’s functional integration demands and the adaptability required for interfacing with diverse external equipment. The electrode array is organized around a central rectangular recording region measuring 1 × 1.5 mm. Within this area, 32 electrodes are arranged into 2 clusters, each comprising a 2 × 8 grid, with a center-to-center spacing of 200 μm. This configuration achieves high spatial resolution while minimizing signal cross talk among electrodes. The electrodes possess a base diameter of 100 μm and are fabricated with a stepped profile to form a conical geometry. The lead traces narrow to 50 μm within the central recording zone and expand to 150 μm outside this area, optimizing spatial efficiency and enabling dense electrode integration within the constrained recording region. The wider leads beyond the central zone reduce electrical resistance during signal transmission, thereby facilitating rapid and low-loss transfer of electrical signals to the external acquisition system. Each electrode is individually wired to the external acquisition apparatus, allowing for independent recording of electrical signals.

**Fig. 1.**
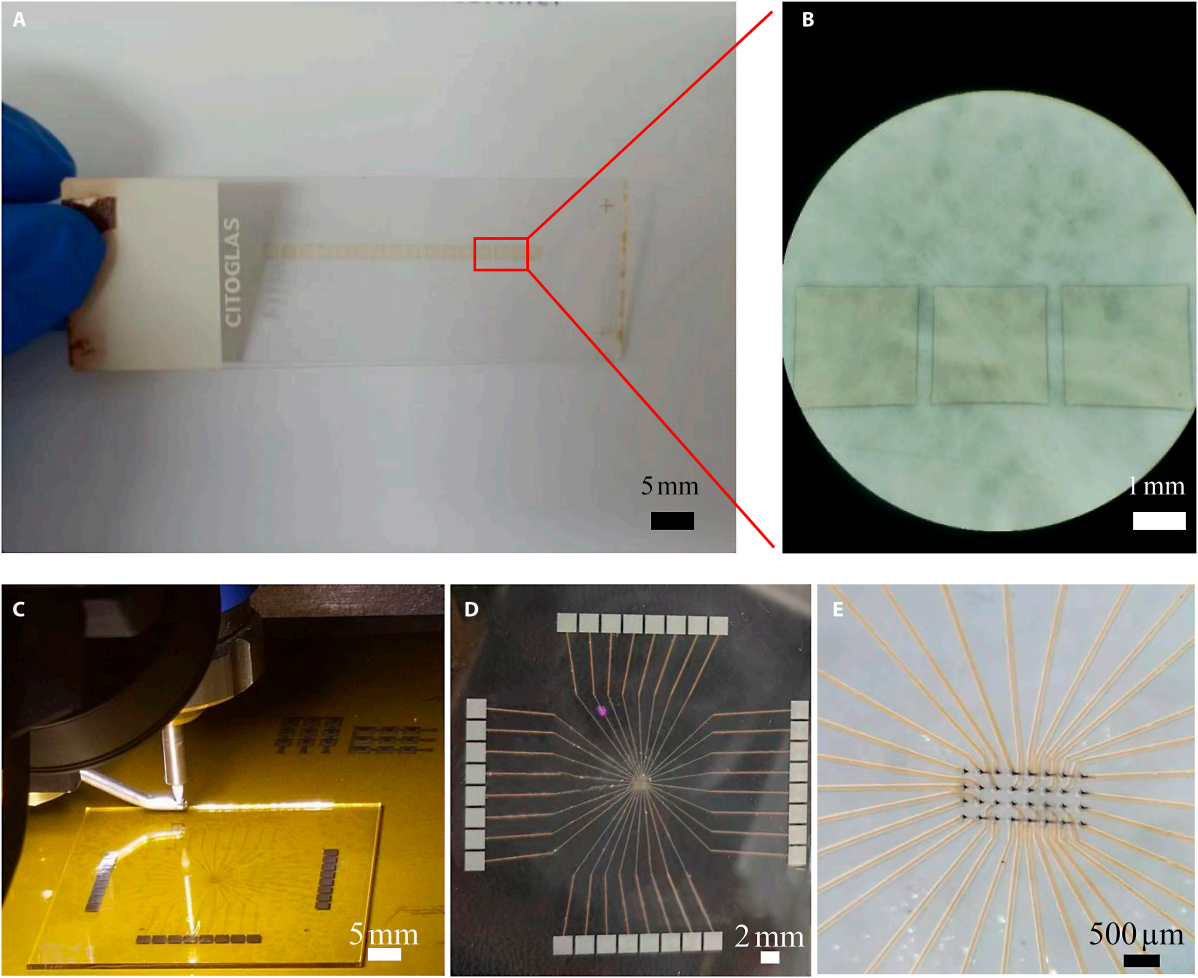
Fabrication and composition of 3-dimensional microelectrode array (3D MEA) chips. (A) Printed polyimide samples on slides with (B) enlargements. (C) 3D MEA chip fabrication process. (D) Full view of the 3D MEA chip. (E) Illustration of the central electrode region of the 3D MEA.

This research utilizes silver nanoparticles as the conductive electrode material owing to their outstanding electrical conductivity and favorable biocompatibility. Polyimide, recognized for its exceptional mechanical strength, thermal stability, and chemical resistance, is employed as the insulating material. To assess the adhesion properties between these materials and the substrate, an aerosol jet printing technique was applied to deposit a polyimide solution onto pretreated glass slides, creating a square array with side lengths of 1 mm (Fig. 1A and B). Optical microscopy analysis demonstrated the presence of uniformly spaced structural units characterized by precisely controlled dimensions. Throughout the fabrication process, the optimization of printing parameters—including print speed, shutter response time, and the number of layers—combined with controlled curing conditions such as heating effectively inhibited adhesion between adjacent structures. The resulting interfaces exhibited strong bonding without any material delamination, thereby fully satisfying the criteria necessary for subsequent MEA fabrication. This dependable fabrication methodology provides a solid foundation for the development of high-performance neural electrodes.

Subsequently, silver electrode arrays were fabricated on glass substrates employing a stepwise deposition technique. Initially, silver was deposited onto the pretreated glass surface in a controlled thickness gradient using high-precision aerosol jet 3D-printing technology (Fig. 1C and D), resulting in a 3D electrode structure with an average height of approximately 200 μm. Following this, conductive lines and peripheral square marking patterns were printed, with wire widths precisely maintained at 50 and 150 μm to ensure stable signal transmission (Fig. 1E). To improve the device’s reliability and durability, an additional polyimide layer, approximately 5 μm thick, was applied over the conductive lines as an insulating layer, conferring superior dielectric properties and mechanical robustness.

**Fig. 2.**
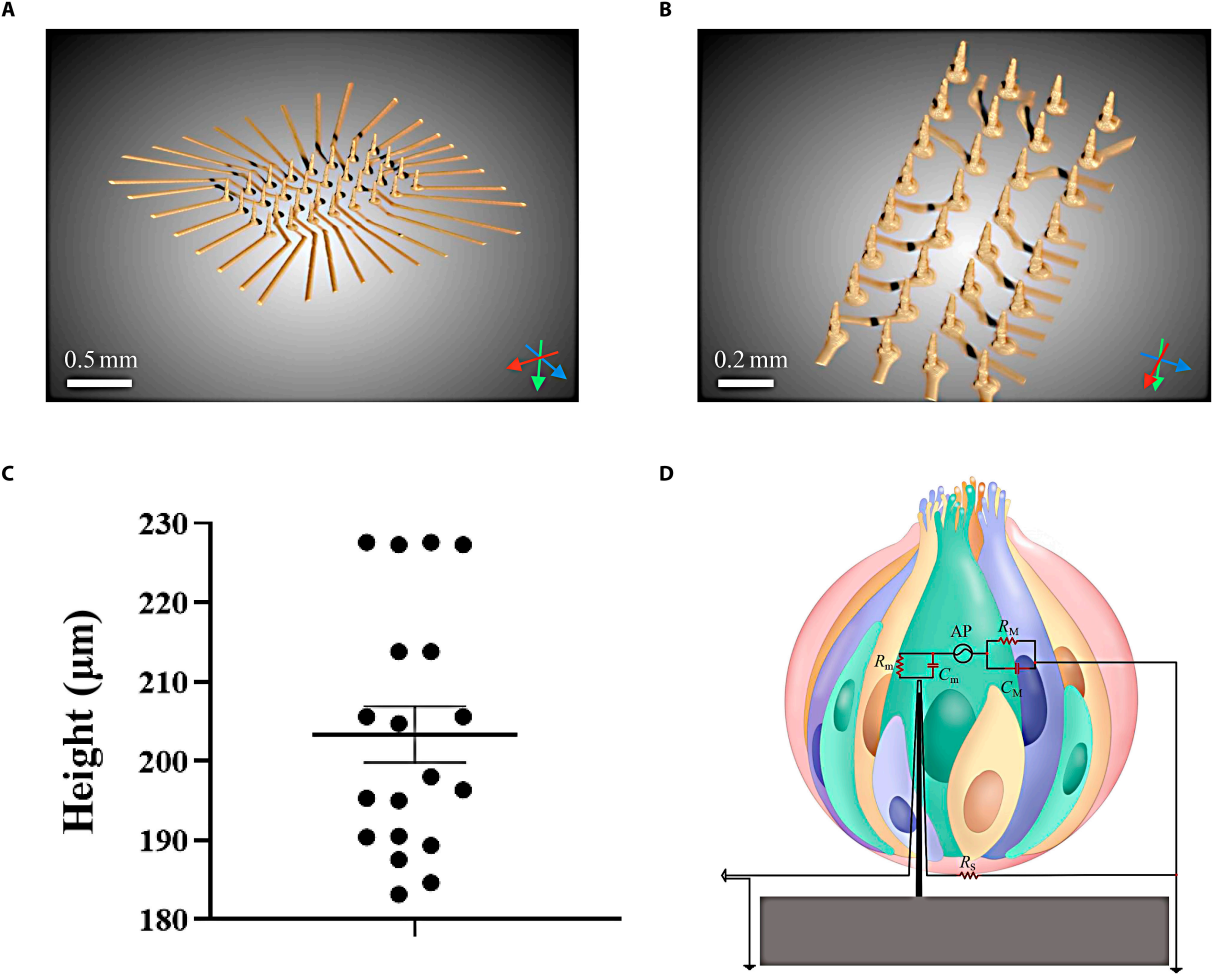
Electron computed tomography (CT) images of a 3-dimensional microelectrode array (3D MEA) chip at different angles and equivalent circuit modeling of taste bud organoids coupled to a 3D MEA chip. Experimental data are expressed as mean ± standard error of the mean (SEM), *n* = 19. (A) Overall CT image of the 3D MEA chip. (B) CT image of the electrode area of the 3D MEA chip. (C) 3D MEA chip microelectrode height statistics results. (D) Equivalent circuit modeling of taste bud organoids coupled to a 3D MEA chip.

Figure 2A and B depict a 3D structural representation of the fabricated chip electrode region, acquired through computed tomography. From a top-down viewpoint, the electrodes are arranged in a uniform grid pattern, with a center-to-center spacing of 200 μm between adjacent electrodes. The positioning accuracy exhibits an error margin of less than ±2 μm, and each electrode demonstrates excellent geometric uniformity. These results further confirm the structural integrity of the electrodes in 3 dimensions, revealing complete isolation between electrodes without any short circuits. This precise electrode configuration enhances spatial resolution for detection and improves the accuracy of signal acquisition, thereby providing a robust foundation for future applications in bioelectric signal detection. Following sample preparation, the microelectrode area is identified using a confocal microscope. A minimum of 4 microelectrodes per row are selected for individual measurement. Selecting a specific electrode isolates it within the viewing field. Subsequently, surface topography images are captured and imported into specialized image processing software (Leica MAP) for detailed analysis. The microelectrode height measurements, as shown in Fig. 2C, indicate that the average height of the microelectrodes on the 3D MEA chip is 203.3 μm.

To date, the majority of reported MEAs have been limited to detecting extracellular signals owing to their planar configuration. Consequently, these devices exhibit a lower signal-to-noise ratio (SNR) relative to intracellular recording techniques such as the whole-cell patch clamp method. To overcome this limitation, 3D stereoscopic electrodes have been developed as an innovative approach for intracellular recording. Several studies have demonstrated that 3D electrodes are capable of successfully capturing intracellular signals [26]. By integrating this chip with taste bud organoids, an equivalent circuit model can be developed, as illustrated in Fig. 2D. The electrochemical interfaces of the electrode, configured either intracellularly or extracellularly, give rise to distinct equivalent circuits contingent upon the specific components present at the cell/electrode interface. In this model, *R*_m_ and *C*_m_ correspond to the resistance and capacitance of the electrode within the intracellular milieu, respectively, while *R*_M_ and *C*_M_ represent the characteristic resistance and capacitance of the cell itself, respectively. *R*_s_ signifies the resistance at the interface between the electrode and the cell membrane, encompassing the sealing resistance. Additionally, the intracellular and extracellular currents generated by the propagation of action potentials are modeled as an alternating current power source, denoted as AP.

**Fig. 3.**
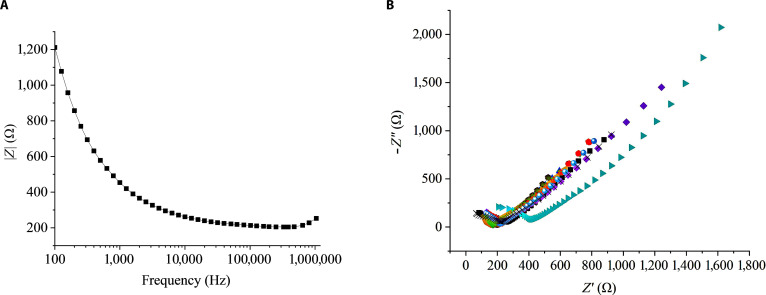
3-dimensional microelectrode array (3D MEA) chip impedance test results. *N* ≥ 30. (A) 3D MEA electrode impedance results at different frequencies. (B) 3D MEA impedance spectral Nyquist plots.

To evaluate the electrical performance of the chip, impedance measurements were performed. Impedance, a fundamental parameter representing the chip’s opposition to alternating current, directly impacts its functionality in applications including bioelectric signal detection [27]. The findings depicted in Fig. 3A indicate that the chip demonstrates an impedance magnitude of approximately 1,200 Ω at a characteristic frequency of 100 Hz. Figure 3B illustrates the Nyquist plot of the electrode impedance spectrum, where the abscissa corresponds to the real component of impedance and the ordinate represents the negative imaginary component. Distinct colors denote different electrodes. Reduced impedance values are indicative of more efficient charge transfer and diminished energy dissipation during electrical interactions with biological tissues, thereby mitigating signal attenuation and distortion. This advantageous performance is attributed to the chip’s unique microelectrode architecture and material selection. In particular, the stepped conical geometry of the electrodes, in conjunction with silver nanoparticle contact pads, enhances the effective contact area with biological tissue, resulting in decreased contact resistance. Furthermore, the polyimide insulating layer provides superior electrical isolation, preventing signal leakage and thereby further improving the chip’s microelectrical properties.

### Measurement of taste bud organoids using 3D MEA

**Fig. 4.**
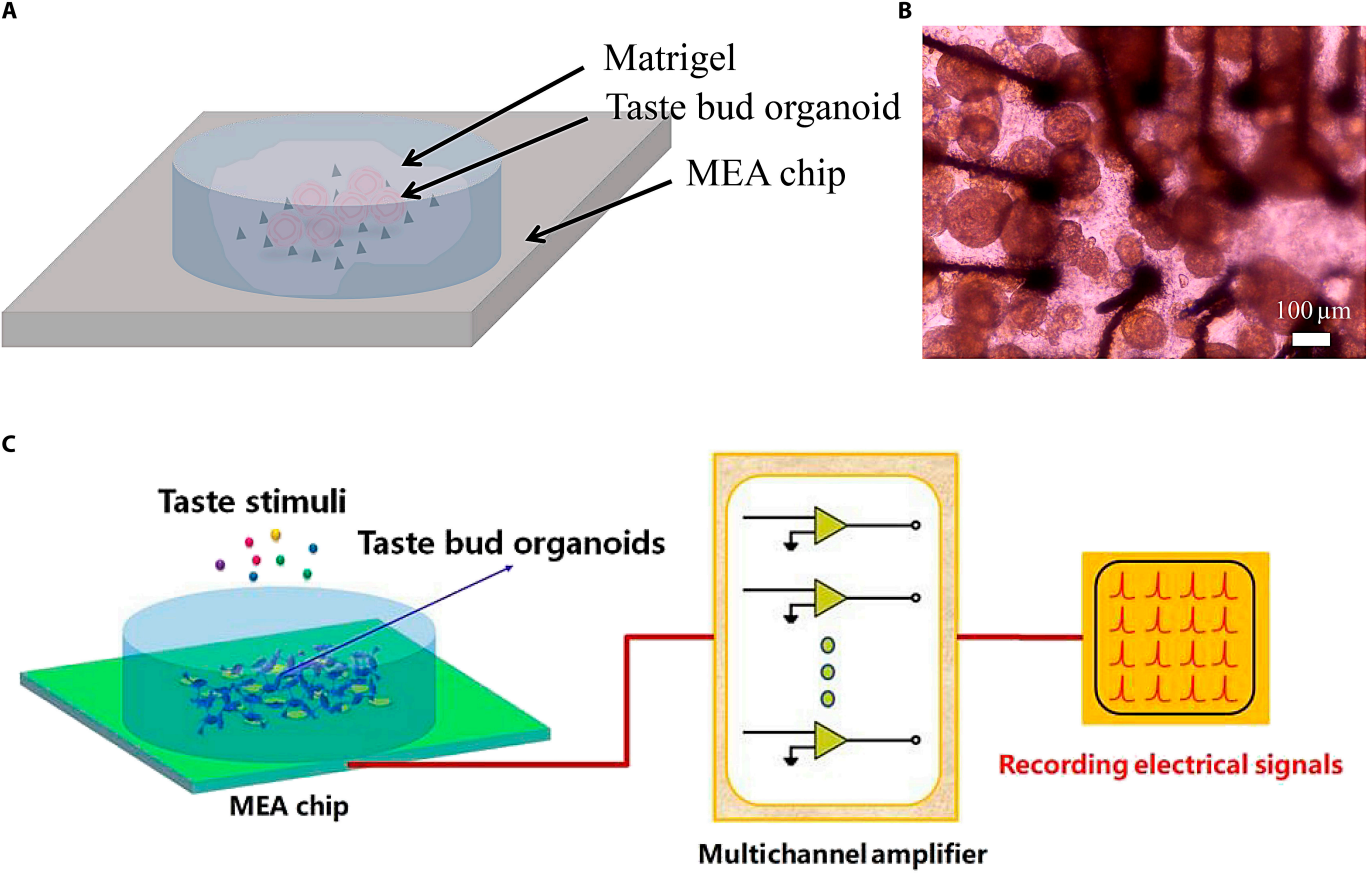
Schematic diagram and system composition of a bionic taste sensor based on a 3-dimensional microelectrode array (3D MEA) chip. (A) Schematic diagram of the coupling of taste bud organoids and a 3D MEA chip. (B) Examples of growth of taste bud organoids inoculated with 3D MEAs. (C) System composition of a bionic taste sensor based on a 3D MEA chip.

A novel 3D taste detection system was developed by integrating 3D MEA chips with taste bud organoids (Fig. 4A and B), which was characterized in our previous work [24]. Briefly, taste bud organoids were harvested and stained by immunofluorescence staining to observe the expression level of the taste biomarkers. The external contours and inner structure were obtained via an optical microscope and a transmission electron microscope. The critical component of this system involves positioning in vitro-cultured taste bud organoids, pretreated with 100 μM caffeine, onto the electrode array of the 3D MEA chip. This configuration leverages the chip’s high spatial resolution to enable real-time monitoring of the electrophysiological activity of the taste bud organoids.

During the experimental procedure, the detection platform was initially stabilized for 30 min under controlled culture conditions of 37 °C and 5% CO_2_. Subsequently, various taste solutions were administered via a pipette to stimulate the organoids. Electrophysiological recordings of the taste bud organoids’ firing activity were obtained using a high-gain, low-noise bioelectrical signal amplifier with a bandwidth of 1 to 3,000 Hz. The sampling frequency was maintained at 30 kHz to ensure high signal fidelity (Fig. 4C). Quantitative analysis of the firing patterns, including metrics such as firing frequency and amplitude, was conducted to assess the impact of caffeine on the taste response functionality of the taste bud organoids.

**Fig. 5.**
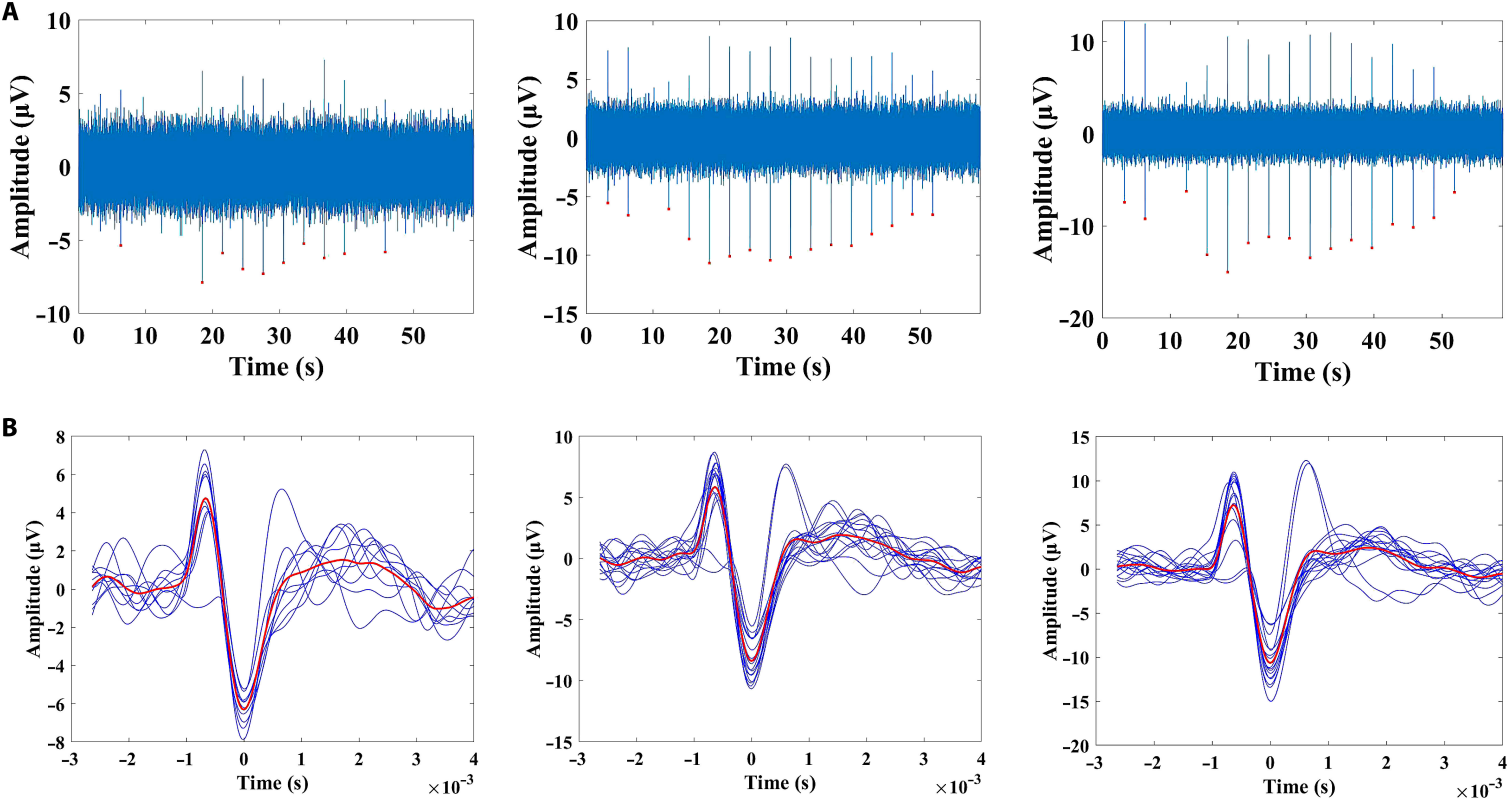
Characterization of spontaneous peak potentials of taste bud organoids detected by the 3-dimensional microelectrode array (3D MEA). (A) Example of spontaneous firing of taste bud organoids from 3 different channels in one experiment. (B) Waveform clustering of the 3 channels in (A).

As illustrated in Fig. 5, the filtered spontaneous firing signals derived from taste bud organoids demonstrate consistent and stable properties, suggesting that the cells within the organoids maintain regular electrophysiological activity on the 3D MEA chip. This stable firing pattern establishes a dependable basis for subsequent signal analysis.

**Fig. 6.**
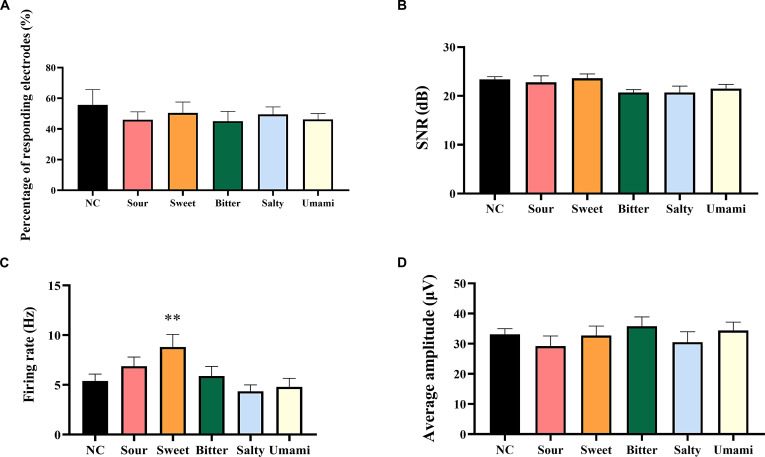
Evaluation of 3-dimensional microelectrode array (3D MEA) electrode performance and quantitative analysis of taste bud organoids’ firing activity. Experimental data are expressed as mean ± standard error of the mean (SEM), *n* = 5, **P < 0.01, number of electrodes used for measurements ≥30, and one-way analysis of variance (ANOVA) accompanied by Dunnett’s multiple comparison test. (A) Percentage of electrode response during taste stimulation of taste bud organoids detected by the 3D MEA. (B) Electrode signal-to-noise ratio in 3D MEA detection of taste stimuli in taste bud organoids. (C) Quantitative analysis of the firing frequency of taste bud organoids under different flavor stimuli. (D) Quantitative analysis of the firing amplitude of taste bud organoids under different flavor stimuli.

Subsequently, the bionic taste sensor was systematically evaluated to characterize its response properties. As illustrated in Fig. 6A, continuous electrode response data were recorded both prior to and following exposure to the 5 basic taste stimuli. Statistical analysis of valid experimental data indicated that the average baseline response rate of the electrode array was 56%. Upon exposure to sour, sweet, bitter, salty, and umami stimuli, the respective response rates for each channel were 46%, 50%, 45%, 50%, and 46%. SNR values were measured at 23.38 in the absence of stimulation and 22.79, 23.62, 20.71, 20.68, and 21.51 for sour, sweet, bitter, salty, and umami stimuli, respectively (Fig. 6B).

To further elucidate the response dynamics of taste bud organoids to various flavor stimuli using the 3D MEA, both the firing rate (Fig. 6C) and firing amplitude (Fig. 6D) were quantified across all active channels. One-way analysis of variance (ANOVA) revealed that taste bud organoids exhibited distinct electrophysiological activity patterns contingent upon the flavor stimulus. Sweet taste elicited the most pronounced response, with a firing frequency of 8.81 Hz, followed by sour taste at 6.88 Hz. Responses to bitter (5.88 Hz), salty (4.36 Hz), and umami (4.79 Hz) stimuli were comparable to the baseline prestimulation frequency of 5.39 Hz. The amplitudes of action potentials remained consistent with baseline measurements, with firing amplitudes recorded as 33.13 μV (no stimulus), 29.23 μV (sour), 32.74 μV (sweet), 35.81 μV (bitter), 30.51 μV (salty), and 34.39 μV (umami), respectively.

### Evaluation of the effects of caffeine on various taste sensing

**Fig. 7.**
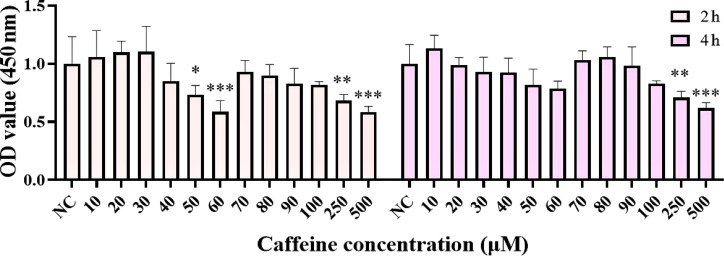
Cytotoxicity test results of caffeine. Experimental data are expressed as mean ± standard error of the mean (SEM), *n* = 5, *P < 0.05, **P < 0.01, ***P < 0.001, and 2-factor method analysis accompanied by Sidak’s multiple comparison test.

To examine the impact of caffeine on taste response, a validation experiment was conducted. Initially, the optimal caffeine concentration for treating taste bud organoids was determined by exposing the organoids to a range of caffeine concentrations spanning from 10 to 500 μM (Fig. 7). A preliminary assessment identified the effective concentration range, with doses below 100 μM tested in 10 μM increments, alongside higher concentrations of 250 and 500 μM. Cellular activity was monitored dynamically at multiple time points (0.5, 1, 2, 4, and 8 h), revealing no significant alterations in activity prior to 2 h at any concentration. However, at the 2-h mark, concentrations of 50, 60, 250, and 500 μM induced a pronounced decrease in cellular activity. Considering this acute response observed at 2 h and the study’s emphasis on the long-term effects of caffeine, a concentration of 100 μM assessed at 4 h was selected for subsequent experiments, as cellular activity remained relatively stable at approximately 83.5% under these conditions.

**Fig. 8.**
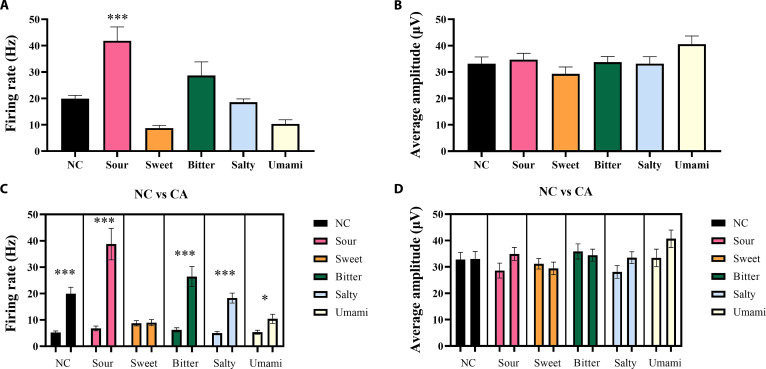
Assessment of the effect of caffeine on taste bud organoids. Experimental data are expressed as mean ± standard error of the mean (SEM), where *n* = 6, number of channels used for statistical analysis ≥ 30, *P < 0.05, ***P < 0.001, and one-way analysis of variance (ANOVA) accompanied by Dunnett’s multiple comparison test. (A) Quantitative statistical results of the average firing rate of caffeine-pretreated taste bud organoids’ flavor response detected by the 3-dimensional microelectrode array (3D MEA). (B) Quantitative statistical results of the mean firing amplitude of caffeine-pretreated taste bud organoids’ flavor response detected by the 3D MEA. (C) Results of the quantitative comparison of the mean firing rates of taste bud organoids’ flavor responses before and after caffeine pretreatment detected by the 3D MEA. (D) Quantitative comparison of the mean firing amplitude of taste bud organoids before and after caffeine pretreatment detected by the 3D MEA.

To examine the variations in firing rate (Fig. 8A) and firing amplitude (Fig. 8B) of caffeine-pretreated taste bud organoids subjected to different flavor stimuli, all available recording channels were subjected to statistical analysis across 5 distinct flavor conditions. One-way ANOVA revealed that the type of flavor stimulus exerted a significant effect on the electrical activity parameters of the taste bud organoids. Notably, the sour stimulus evoked the most robust response, with an average firing frequency of 41.85 Hz, followed by the bitter stimulus at 28.67 Hz. In contrast, responses to salty (18.57 Hz), umami (10.32 Hz), and sweet (8.76 Hz) stimuli were lower than the prestimulus baseline firing rate of 19.91 Hz. The amplitude of action potentials did not differ significantly from baseline measurements.

A comparative analysis of neuronal firing activity before and after caffeine administration revealed that taste bud organoids pretreated with caffeine exhibited significantly elevated spontaneous firing rates in the absence of taste stimuli (5.39 Hz versus 19.91 Hz; see Fig. 8C). This finding implies that caffeine may modulate fundamental gustatory signaling by enhancing the baseline excitability of these organoids. Upon exposure to sour, bitter, salty, and umami stimuli, the spontaneous firing frequencies of caffeine-pretreated taste bud organoids were consistently and significantly higher than those observed in the decaffeinated control group. The most pronounced difference was noted during sour stimulation (6.88 Hz versus 41.85 Hz), followed by bitter (5.88 Hz versus 28.67 Hz), salty (4.36 Hz versus 18.57 Hz), and umami (4.79 Hz versus 10.32 Hz) stimuli. Notably, firing rates during sweet stimulation were comparable between the 2 groups (8.81 Hz versus 8.76 Hz). Furthermore, measurements of firing amplitude indicated that, during sour, salty, and umami taste stimulations, caffeine-pretreated organoids exhibited marginally higher amplitudes relative to the decaffeinated group (29.23 μV versus 34.67 μV, 30.51 μV versus 33.20 μV, and 34.39 μV versus 40.58 μV, respectively); however, these differences did not reach statistical significance (refer to Fig. 8D).

These findings demonstrate that taste bud organoids possess intrinsic electrical activity in the absence of external stimuli, reflecting their inherent electrophysiological state or “baseline excitability”. Following caffeine pretreatment, the spontaneous firing frequency significantly increased from 5.39 to 19.91 Hz, indicating a pronounced enhancement. This observation strongly suggests that caffeine facilitates the depolarization of the cell membranes within taste bud organoids, thereby promoting the generation of electrical signals. Caffeine, an alkaloid known to modulate various intracellular signaling pathways—such as inhibiting phosphodiesterases [28], which elevates cyclic adenosine monophosphate levels, or directly acting on intracellular calcium-release channels like ryanodine receptors [29]—results in increased intracellular calcium ion concentrations. These biochemical effects can alter membrane potential and cellular excitability, thereby increasing the likelihood of action potentials or analogous firing events. Consequently, caffeine influences ion channels or signaling cascades in taste bud organoid cells, rendering them more excitable.

Moreover, the caffeine-pretreated group exhibited elevated firing frequencies in response to sour, bitter, salty, and umami taste stimuli, indicating that these organoids generated stronger and more frequent electrical signals upon exposure to these tastants. This pattern reflects an enhanced or heightened sensitivity to these specific taste modalities. Given that caffeine raises baseline excitability, the taste stimulus-induced signals are superimposed upon this elevated baseline, culminating in an overall increase in firing frequency. It is important to note that different taste modalities are mediated by distinct molecular mechanisms; for instance, bitter and umami tastes typically involve G protein-coupled receptors and downstream signaling pathways, whereas sour and salty tastes are often transduced via ion channels. Caffeine may selectively potentiate or modulate certain pathways, such as by influencing calcium ion release or phosphodiesterase activity, thereby amplifying signals elicited by these particular stimuli. Additionally, considering that caffeine itself is a bitter compound, it may interact with bitter taste receptors, thereby affecting bitter taste signaling. The absence of substantial effects on sweet taste responses further supports the notion that caffeine’s modulatory effects are selective, predominantly targeting mechanisms associated with sour, bitter, salty, and umami taste transduction while exerting minimal influence on sweet taste pathways.

**Fig. 9.**
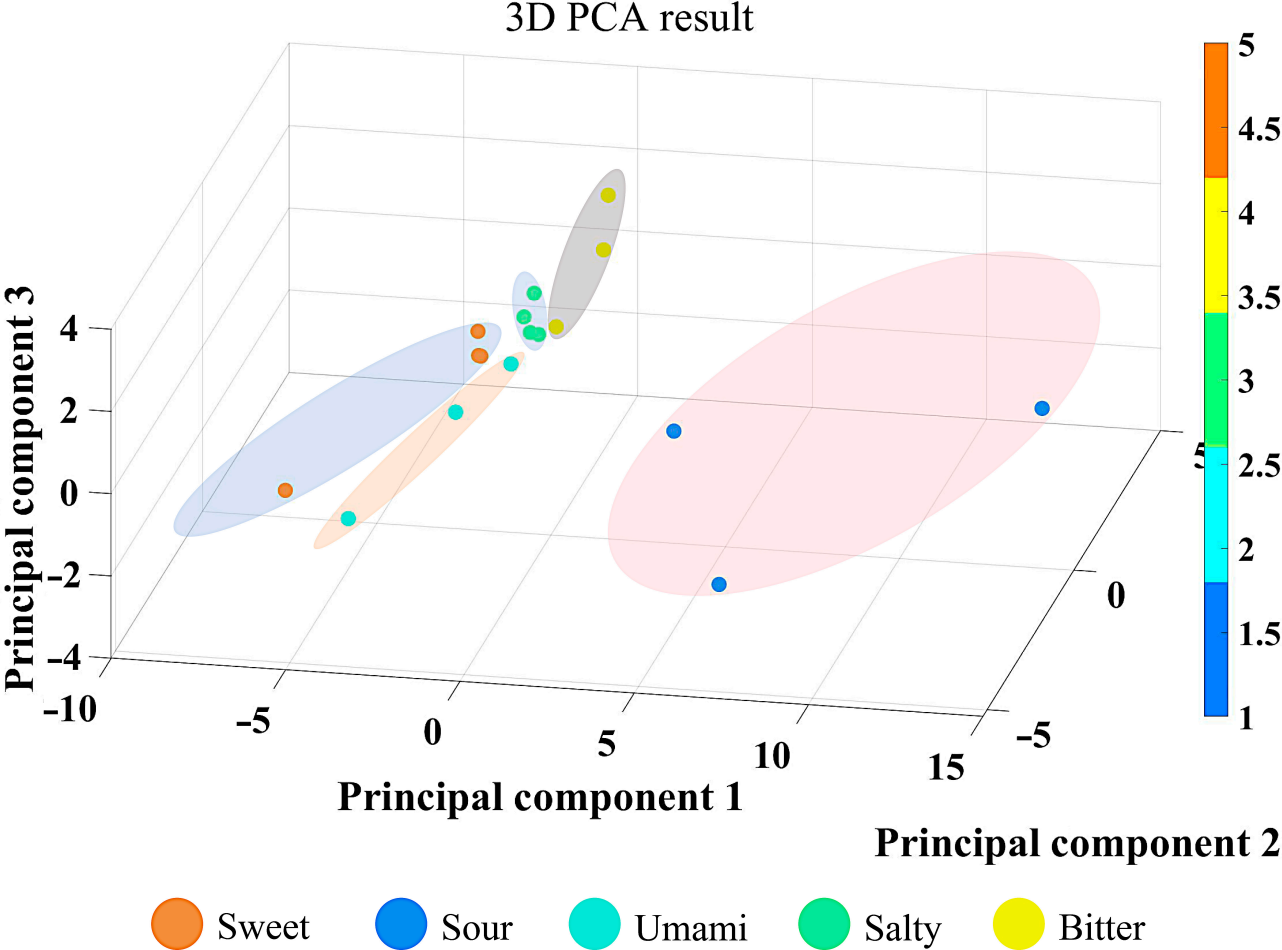
Projections of the principal component analysis (PCA) of the difference in firing rates of caffeine-treated taste bud organoids in response to 5 flavor stimuli on the first 3 principal components.

The data were subsequently subjected to principal component analysis (PCA) for dimensionality reduction to distinguish the responses of caffeine-treated taste bud organoids to various flavor stimuli (Fig. 9). In each of the 5 experimental trials, the change in mean firing rate (ΔMFR′) induced by each flavor stimulus was treated as a sample set, with responses recorded across *n* channels representing *n* dimensions (where *n* ≥ 15). The first 3 principal components derived from the sample sets across the 5 experiments were projected, revealing that responses corresponding to 5 distinct taste categories (sour, sweet, bitter, salty, and umami) formed 5 separate and independent clusters. These 3 principal components collectively accounted for 90.72% of the total variance (with 65.28%, 15.49%, and 9.95% attributed to PC1, PC2, and PC3, respectively; *n* = 5), indicating that the reduced-dimensional representation effectively captured the original taste response patterns with high fidelity. These results demonstrate that the taste detection platform can discriminate the unique peak potential emission patterns of caffeine-treated taste bud organoids in response to sour, sweet, bitter, salty, and umami stimuli. Consequently, the 3D MEA chip-based biomimetic taste sensor presented herein is capable of detecting and differentiating the 5 basic tastes based on the peak potential response patterns of caffeine-treated taste bud organoids, thereby confirming the multidimensional separability of taste response profiles.

## Conclusion

This study presents a novel biomimetic taste biosensor that integrates, for the first time, taste bud organoids with a 3D MEA. Unlike conventional planar MEAs, the 3D MEA features vertically structured electrodes (about 20 μm in height), which better match the 3D architecture of taste bud organoids, thereby enhancing SNR and recording stability. Utilizing these sensors, the research systematically assessed the responses of caffeine-treated taste bud organoids to various flavor stimuli, thereby achieving accurate differentiation and quantitative analysis of the 5 fundamental taste modalities. This work not only advances the development of organoid-based bioelectronic platforms for gustatory sensing but also provides new insights into the molecular and cellular mechanisms by which caffeine modulates taste perception, with potential applications in food science, drug screening, and sensory biology.

Furthermore, 3D electrode arrays fabricated via vertical stacking or thermoforming techniques demonstrate the capability to record electrophysiological signals at multiple depths within 3D biological tissues, including neurospheres and cardiac organoids [30]. For instance, a high-density 3D MEA comprising 26,400 electrodes distributed over a 4 × 2 mm^2^ area is capable of capturing subcellular activities, such as axonal conduction, thereby substantially enhancing the spatial resolution of signal acquisition [31]. Enhanced accuracy in spike classification can be achieved by ascertaining the spatial position of the active neuron, thereby enabling the recognition of spike waveforms generated by more distant cells with lower signal amplitudes [30]. Flexible substrates, including polyimide and liquid crystal polymers, in conjunction with poly(3,4-ethylenedioxythiophene):poly(styrene sulfonate) conductive coatings, are employed to reduce implantation-induced damage and enhance electrode–tissue conformity. For instance, a flexible implantable MEA developed at Zhejiang University demonstrated successful long-term monitoring of electrical activity within a 3D myocardial spheroid, achieving a 2.69-fold improvement in signal stability. Furthermore, 3D MEA chips integrated with microfluidic channels enable localized delivery of pharmacological or biochemical agents while concurrently recording neural network responses, thereby offering dynamic data valuable for drug screening and toxicity evaluation [25]. Concerning the 3D MEA chip utilized in this study, future research will focus on implementing electrode modifications and enhancements informed by recent developments to further improve the sensor’s detection performance. In the experimental setup, we will incorporate the detection of mixed tastes, extract response signals from taste bud organoids, and use analytical methods such as machine learning to calculate additional parameters, thereby comprehensively elucidating the response mechanisms of taste bud organoids.

## Methods

### Design and fabrication of 3D MEA chips

A substrate composed of Schott BF33 borosilicate glass, measuring 50 mm in edge length, was employed for the fabrication of the 3D structure of the MEA chip. The entire manufacturing process was conducted by Beijing YUNS Technology Company. During fabrication, a high-precision aerosol jet 3D-printing technique was utilized to deposit silver material incrementally onto the surface of the pretreated glass substrate, thereby creating a 3D electrode structure with an average height of approximately 200 μm. Following this, conductive circuits and peripheral square contact pads were printed. To improve the device’s reliability and durability, an insulating layer of polyimide film, approximately 5 μm thick, was subsequently applied over the wire surfaces.

### Height and impedance measurement of 3D MEA electrodes

For the measurement of electrode height, following sample preparation, a confocal microscope was employed to precisely localize the electrode regions. More than 4 electrodes per row were selected for individual imaging. The acquired morphological images were subsequently imported into the Leica MAP software for analysis. Initially, background tilt was corrected using least squares leveling, and high-frequency noise was attenuated through digital filtering. Morphological analysis was then conducted utilizing 3 × 3 pixel structural elements combined with 20% to 80% grayscale thresholding. Processed images were extracted from 3D profile data along the centerline path, and 3D reconstruction was performed to generate morphology views observable from multiple perspectives. Finally, electrode heights were quantified using the height measurement tool, and statistical representations of the data were produced.

The MEA chip was mounted within an electrochemical testing setup, with each of the 3 electrodes individually connected to a CHI600E electrochemical workstation. To ensure a stable reference potential and accurate measurements, an Ag/AgCl electrode was employed as the reference electrode, while a platinum wire electrode served as the counter electrode. The platinum wire electrode, characterized by its large surface area and excellent electrical conductivity, effectively completes the circuit and facilitates current transfer. Prior to experimentation, the platinum wire electrode surface was cleaned to remove contaminants. Additionally, the potassium chloride solution within the Ag/AgCl electrode was verified to be sufficient to maintain stable performance. The ac voltage amplitude was set at 10 mV with a dc bias of 0 V, and measurements were recorded at 10 points per decade. Frequency scanning was conducted from low to high frequencies. Upon confirmation of correct connections and parameter settings, the test was initiated. The instrument automatically scanned the specified frequency range, recording impedance data including impedance magnitude (|*Z*|) and phase angle (*θ*). Upon completion, the software generated impedance spectra such as Nyquist and Bode plots. These data were exported and further analyzed using software such as Origin to plot impedance as a function of frequency, thereby facilitating evaluation of the microelectrodes’ performance.

### Caffeine concentration screening and pretreatment of taste bud organoids

Caffeine was initially dissolved in phosphate-buffered saline (PBS) to prepare a 10 mM stock solution for storage purposes. Taste bud organoids, cultured for 10 d, were seeded into 96-well plates at a density of 200 organoids per well and incubated at 37 °C with 5% CO_2_ for 24 h. Subsequently, caffeine was administered to achieve final concentrations of 0, 10, 20, 30, 40, 50, 60, 70, 80, 90, 100, 250, and 500 μM, encompassing the range of typical daily human intake, including concentrations representative of coffee consumption (approximately 400 to 600 μM), extending up to subtoxic levels. Following an additional 48-h incubation period, cellular viability was assessed at 0.5, 1, 2, 4, and 8 h using the Cell Counting Kit-8 assay. For electrophysiological analyses, caffeine-treated organoids were cultured for 10 to 14 d, with caffeine added to the culture medium at the time of passaging to a final concentration of 100 μM to achieve the maximal stimulatory effect while minimizing the impairment of cell viability, thereby enabling a more effective analysis of caffeine’s influence on taste transduction.

### Electrical signal acquisition methods

In this investigation, a 60-channel MEA chip was employed alongside an RHS2000 system for data acquisition, operating at a sampling frequency of 30 kHz and a bandwidth spanning 1 to 3,000 Hz. This configuration enabled the effective recording of neural action potentials as well as other electrophysiological activities of neurons. Prior to electrical signal measurement, the culture medium was removed from the culture chamber and the chamber was rinsed with PBS. The flavor substance utilized for the measurements was also dissolved in PBS. Given that consistent contact between all electrodes and the organoid could not be assured in each experimental trial, signals from all electrodes were recorded and stored in a designated file format. Subsequently, these data were imported into the MATLAB software for analysis and processing, facilitating the appropriate filtering of erroneous signals characterized by either lack of response or excessive noise.

### Data analysis

To facilitate interoperability with various neurophysiological data analysis platforms, the system incorporates the Intan NEX Converter software, enabling the transformation of raw data into NEX format files. These files are readily compatible with specialized neurophysiological analysis applications such as Offline Sorter and Spike2, allowing for comprehensive examination and spike sorting to investigate neuronal firing patterns. This flexible data processing approach markedly enhances the efficiency and convenience of data analysis workflows. Additionally, electrophysiological signals were digitally filtered using a Butterworth filter implemented in MATLAB to isolate peak potential signals (spikes) within the 200- to 3,000-Hz frequency range [32]. The experimental data are expressed as the mean value accompanied by the standard error of the mean.

### Analysis method of peak potential signals

The multichannel signals underwent high-pass filtering to detect peak potentials, employing a threshold established at 5 times the standard deviation of the neural signal. Upon identification of a peak potential, the corresponding timestamps for each unit’s peak potential occurrence were documented, enabling the subsequent computation of the frequency of these peak potential events [32]. In taste bud organoids not subjected to caffeine stimulation, spontaneous and poststimulation firing rates were quantified by calculating the MFR prior to and following taste stimulation. The taste response was evaluated by the difference between the MFR before and after stimulation (ΔMFR), which was defined as follows:

$$\begin{array}{c} \text{ΔMFR}=\mathrm{MFR}\;\left(\text{after taste stimulation}\right)\\ -\mathrm{MFR}\;\left(\text{before taste stimulation}\right) \end{array}$$ (1)

In the study involving taste bud organoids exposed to caffeine, the taste response was assessed by initially determining the mean firing rate MFR1 (pre) prior to taste stimulation and MFR1 (post) following the onset of stimulation within the control group. The change in firing rate (ΔMFR1) was then calculated as follows:

$$\begin{array}{c} \text{ΔMFR}1=\mathrm{MFR}1\;\left(\text{after taste stimulation}\right)\\ -\mathrm{MFR}1\;\left(\text{before taste stimulation}\right) \end{array}$$ (2)

Subsequently, the mean firing rate (MFR2) of the caffeine-treated group was computed for both pre- and posttaste stimulation periods. The difference between these rates (ΔMFR2) served as a measure to assess the taste response in the taste bud organoids influenced by caffeine.

$$\begin{array}{c} \text{ΔMFR}2=\mathrm{MFR}2\;\left(\text{after taste stimulation}\right)\\ -\mathrm{MFR}2\;\left(\text{before taste stimulation}\right) \end{array}$$ (3)

The ultimate disparity in firing rates between the 2 cohorts, indicative of caffeine’s impact on the gustatory response, was determined as follows:

$${\text{ΔMFR}}^{\prime }=\text{Δ}\mathrm{MFR}2-\text{Δ}\mathrm{MFR}1$$ (4)

Statistical analyses of MFR, ΔMFR, and ΔMFR′ elicited by various flavors were conducted utilizing the GraphPad Prism software. Furthermore, waveforms associated with peak potentials were examined through clustering and PCA implemented in MATLAB. Channels displaying signal amplitudes greater than 1 mV were excluded from the dataset.

### Principal component analysis

In the PCA, the eigenvariable for each channel was defined as the change in the mean firing rate (ΔMFR) observed before and after stimulation. Given *m* distinct stimulus types, each stimulus was characterized by *n* eigenvalues (where *n* ≥ 3), resulting in a data matrix of dimensions *m* × *n*. This matrix was subsequently subjected to a linear transformation and orthogonalization process to extract the top 3 principal components corresponding to the largest eigenvalues. These 3 principal components served as new eigenvalues, effectively substituting the original *n* eigenvalues and thereby facilitating dimensionality reduction of the dataset [32].

## Data Availability

The experimental data will be made available on request from the corresponding authors.
